# Evaluation of Controlled Vocabulary Resources for Development of a Consumer Entry Vocabulary for Diabetes

**DOI:** 10.2196/jmir.3.3.e24

**Published:** 2001-08-28

**Authors:** Timothy B Patrick, Harpreet K Monga, MaryEllen C Sievert, Joan Houston Hall, Daniel R Longo

**Affiliations:** ^1^Department of Health Management and InformaticsCenter for Family Medicine ScienceUniversity of Missouri-ColumbiaUSA; ^2^Department of Health Management and InformaticsUniversity of Missouri-ColumbiaUSA; ^3^School of Information Science and Learning TechnologyDepartment of Health Management and InformaticsUniversity of Missouri-ColumbiaUSA; ^4^Dictionary of American Regional English ProjectUniversity of Wisconsin-MadisonUSA; ^5^Department of Family and Community MedicineCenter for Family Medicine ScienceUniversity of Missouri-ColumbiaUSA

**Keywords:** Communication barriers, vocabulary, controlled, public health

## Abstract

**Background:**

Digital information technology can facilitate informed decision making by individuals regarding their personal health care. The digital divide separates those who do and those who do not have access to or otherwise make use of digital information. To close the digital divide, health care communications research must address a fundamental issue, the consumer vocabulary problem: consumers of health care, at least those who are laypersons, are not always familiar with the professional vocabulary and concepts used by providers of health care and by providers of health care information, and, conversely, health care and health care information providers are not always familiar with the vocabulary and concepts used by consumers. One way to address this problem is to develop a consumer entry vocabulary for health care communications.

**Objectives:**

To evaluate the potential of controlled vocabulary resources for supporting the development of consumer entry vocabulary for diabetes.

**Methods:**

We used folk medical terms from the Dictionary of American Regional English project to create exended versions of 3 controlled vocabulary resources: the Unified Medical Language System Metathesaurus, the Eurodicautom of the European Commission's Translation Service, and the European Commission Glossary of popular and technical medical terms. We extracted consumer terms from consumer-authored materials, and physician terms from physician-authored materials. We used our extended versions of the vocabulary resources to link diabetes-related terms used by health care consumers to synonymous, nearly-synonymous, or closely-related terms used by family physicians. We also examined whether retrieval of diabetes-related World Wide Web information sites maintained by nonprofit health care professional organizations, academic organizations, or governmental organizations can be improved by substituting a physician term for its related consumer term in the query.

**Results:**

The Dictionary of American Regional English extension of the Metathesaurus provided coverage, either direct or indirect, of approximately 23% of the natural language consumer-term-physician-term pairs. The Dictionary of American Regional English extension of the Eurodicautom provided coverage for 16% of the term pairs. Both the Metathesaurus and the Eurodicautom indirectly related more terms than they directly related. A high percentage of covered term pairs, with more indirectly covered pairs than directly covered pairs, might be one way to make the most out of expensive controlled vocabulary resources. We compared retrieval of diabetes-related Web information sites using the physician terms to retrieval using related consumer terms We based the comparison on retrieval of sites maintained by non-profit healthcare professional organizations, academic organizations, or governmental organizations. The number of such sites in the first 20 results from a search was increased by substituting a physician term for its related consumer term in the query. This suggests that the Dictionary of American Regional English extensions of the Metathesaurus and Eurodicautom may be used to provide useful links from natural language consumer terms to natural language physician terms.

**Conclusions:**

The Dictionary of American Regional English extensions of the Metathesaurus and Eurodicautom should be investigated further for support of consumer entry vocabulary for diabetes.

## Introduction

### The consumer vocabulary problem

Digital information technology can facilitate informed decision making by individuals and can contribute to the realization of 2 important goals of the United States (US) health care establishment. The first of these goals is the US Healthy People 2010 goal of increasing "life expectancy and quality of life . . . by helping individuals gain the knowledge, motivation, and opportunities they need to make informed decisions about their health" [1]. The second goal is the US National Cancer Institute (NCI) goal of improving the "use of high quality, evidence-based cancer communications regardless of race, ethnicity, health status, education, income, age, gender, culture, or geographic region" [2]. It is certainly the case that " [a]t no other time in history has it been so easy for so many people to access such a vast wealth of information" [3], and it is also the case that consumers of health care, particularly laypersons, increasingly use the Web as a source of health care information [4]. The digital divide [5] has been described as the separation of populations into those who do and those who do not have access to or otherwise make use of digital information. The digital divide is problematic for the goals of Healthy People 2010 and NCI.

A promise of health care-communications research is that it can offer solutions to the digital divide. Ensuring universal access to information technology will not, however, be a sufficient solution. To close the digital divide, health care-communications research must address an issue that is more fundamental than the digital divide: the issue that we call the consumer vocabulary problem.

The consumer vocabulary problem is that consumers of health care, at least those who are laypersons, are not always familiar with the professional vocabulary and concepts used by providers of health care and by providers of health care information, and, conversely, health care and information providers are not always familiar with the vocabulary and concepts used by consumers. This bidirectional communication problem is more fundamental than the digital divide because it affects the use of information in all forms, both digital and non-digital. Access to a high speed network and a World Wide Web (Web) browser will serve little purpose for a health care consumer who lacks the vocabulary necessary to: ask questions about his or her health care, search for information to support his or her decisions about it, or understand such information when he or she does manage to access it.

### Vocabulary differences in health are Communications: misunderstandings, outcomes, and information access

Potential misunderstandings attendant upon vocabulary differences in health care communications may reduce the quality of patient-physician interaction, result in poor health outcomes and patient satisfaction, impact consumer access to health care information, and have implications for informed consent.

The effect of vocabulary differences on patient-physician communication has long been recognized and studied by medical anthropologists and practitioners [6-8]. The importance of patient-physician communication for patient satisfaction and health outcomes is increasingly recognized [9] and communication skills are increasingly promoted as a necessary part of medical education [10-12].

Not all problems in patient-physician communication are the result of vocabulary differences. Some communication problems result from differences in values between the patient and the physician [13]. However, the quality of patient-physician communication may be compromised by differences in vocabulary that result in poor health outcomes. The patient's misunderstanding of professional language (or misunderstandings by the patient's caretaker), whether or not due to illiteracy [14], may lead to misunderstandings of crucial diagnostic or treatment information, and this may lead to a lack of proper compliance [15]. Previous studies, for example, have demonstrated a need for close attention to vocabulary differences in the education and treatment of patients with chronic conditions such as diabetes [16,17]and asthma [18-22].

Vocabulary and other language differences may be particularly significant in cross-cultural contexts [23,24]. For example, a physician not familiar with the term *the blood disease* as used by some African Americans to refer to cancer [25], might very easily mistake it for a synonym of *low blood* as used by some African Americans to refer to anemia [8]. For another example, consider *sugar*, *sugar diabetes,* and *diabetes*. A common belief among clinicians in the Midwestern and Southern US is that *sugar* is a term used by rural African Americans to refer to diabetes. However, a recent study [26] suggests that in some cases persons who say they have *sugar* have very different health beliefs regarding their disease than do persons who say they have *sugar diabetes* or *diabetes*. According to [26], persons who said they had *sugar* were "more likely to say that their condition was not serious and was curable." These different health beliefs have clear implications for compliance. If persons who believe they have *sugar* also believe their condition is not serious, they may be less diligent in matters of controlling their blood glucose levels.

With regard to health care-information access, in a previous study [27] we showed that the vocabulary used for the retrieval of health care information on the Web is problematic. In that study, we compared retrieval results for several commonly-used lexical variants, ie, different ways of writing the same term commonly used by laypersons. We found that the variant used clearly influenced the number of items retrieved. But, lexical variants are not the only sort of vocabulary barrier to health care information. In a recent Associated Press (AP) story [28], it was reported that former US Rep Geraldine Ferraro has *blood cancer*. Regardless of whether it was used appropriately in this case, *blood cancer* is a clear example of consumer vocabulary. If recent reports of the increasing use of the Web by consumers to get health care information [4] are accurate, it is likely that the AP story provided an impetus for some consumers to search for related information on the Web. Accordingly, we conducted a search (on June 25, 2001) on www.altavista.com for *blood cancer*. The search returned 2,616 results while a search with *multiple myeloma*(which the AP story used, apparently as a more specific term than *blood cancer)* returned 33,279 results. On www.google.com *,* the difference was 5,450 versus 46,700.The number of items retrieved is not at issue as much as the quality and relevance of the items retrieved [29-32]. Nevertheless, these results do suggest that there may be significant practical differences between consumer vocabulary and professional vocabulary for Web information retrieval.

Vocabulary differences affecting information retrieval and conceptual frameworks for reconciling those differences have long been studied in the fields of information science and informatics [33-36]. The problem of vocabulary differences is complicated for health care consumer informatics due to variations in the precision of the meanings of health care vocabulary as used by laypersons, and the lack of consistent semantic overlap of layperson vocabulary with professional vocabulary [6,35].

The lack of semantic overlap may have implications for informed consent. Perhaps the best-known US example of this is the infamous Tuskegee syphilis study [37]. The lack of informed consent by participants in the Tuskegee study may have been the result of mutual misunderstandings of the term *bad blood* as used by the rural African American subjects and as used by the US Public Health Physicians running the study. According to [37], some subjects of the study were told they had *bad blood*. The US Center for Disease Control (CDC) was in charge of the study in its later stages and an official of the CDC "stated that he understood the term 'bad blood' was a synonym for syphilis in the black community" [37]. However, also according to [37], a surviving subject of the study stated, ""That could be true. But I never heard no such thing. All I knew was that they just kept saying I had the bad blood -- they never mentioned syphilis to me, not even once." [37] . Indeed, such misunderstandings concerning *bad blood* may yet persist. In 1985, after the Tuskegee study had been widely reported in the popular press, *bad blood* was still reported to be a slang or nonstandard dialect synonym for syphilis [8].

### The need for a consumer entry vocabulary for health care communications

The types of problems just described will differ in degree from case to case; some health care consumers are more familiar than others with professional vocabulary, and likewise some health care providers are more familiar than others with consumer vocabulary. In addition, there may be differences between countries, eg, differences between US physicians and British physicians in their familiarity with consumer language. Nevertheless, in general the kind of bidirectional communication problems we are discussing can make it difficult for individuals to get the information they need to support their decisions about their personal health care, and so these problems run counter to the goals of Healthy People 2010 and the US National Cancer Institute (NCI).

One way to address these bidirectional communication problems is to develop a *consumer-entry vocabulary* for health care communications. An entry vocabulary links commonly used terms to terms in some specialized vocabulary [36]. A consumer entry vocabulary for health care communications will link terms familiar to the consumer to possibly-unfamiliar professional terms used to index, describe, and communicate health care information. It will also function as an aid to communication between providers and patients (or their caretakers) [15]. From the point of view of the health care provider, the entry vocabulary will link terms familiar to the provider with commonly used, but perhaps unfamiliar, consumer terms. This bidirectional linking of consumer and professional vocabulary has elsewhere been described as the problem of reconciling groups of sub-languages [34].

### Overview of the study

A controlled vocabulary may be defined minimally as a concept-based vocabulary in which both the concepts and the terms used to express them are subjected to some level of control; only some subset of concepts is expressed, and only some terms and term variations are allowed as expressions of those concepts. Typically, an entry vocabulary will link natural language terms to terms in a controlled vocabulary. Since, however, health communication begins and ends with the natural language of professional practice and that of common sense, we were interested in using a controlled-vocabulary resource to link 2 natural language domains of vocabulary. (We use *vocabulary resource* here to allow for an organized collection of vocabularies, such as the Unified Medical Language System [UMLS] Metathesaurus.) We were *not* interested in questions of how much of a given natural language domain, whether professional or layperson, was contained in the controlled vocabulary resource. No matter how much is invested in controlled vocabulary resources, and no matter how comprehensive they appear, we assume that there will always be some natural residue of current vocabulary, whether professional or layperson, that is not contained in any controlled vocabulary resource, and that it may be associated with bidirectional communication problems in health care. One way to reap the benefits of the investment that has been made in building vocabulary resources may be to use them to provide indirect, though perhaps only approximate, links between natural language domains of vocabulary.

**Figure 1:**
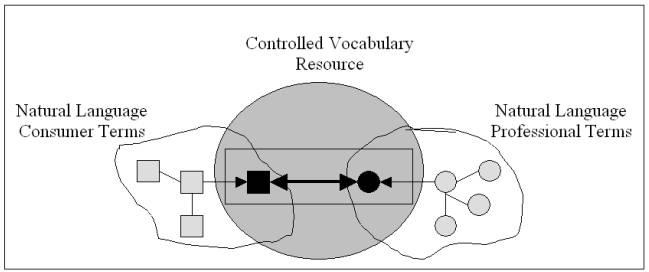
A controlled vocabulary resource providing a bridge between consumer terms and professional terms

By using a controlled vocabulary resource to link 2 natural language domains of vocabulary, we may provide entry vocabulary from one domain to the other. Specifically, in this pilot study we evaluated controlled vocabulary resources for their capacity to support an entry vocabulary from natural language health care consumer vocabulary to natural language health care professional vocabulary. The conceptual model we used is depicted in Figure 1.

As shown in Figure 1, a controlled vocabulary resource (such as the Metathesaurus) may function as a bridge between natural language consumer terms and natural language professional terms. The point of the model is to address the need for linking natural language domains, and depends on there being some overlap between the natural language consumer vocabulary, the controlled vocabulary resource, and the natural language professional vocabulary. This overlap allows us to make use of the controlled vocabulary resource to link the natural language consumer terms to the natural language professional terms. Figure 1 shows 3 natural language consumer terms linked to 4 natural language professional terms.

In Figure 1, assume that the 3 consumer terms are located in the same semantic neighborhood [38], that is, are synonymous, nearly synonymous, or otherwise closely related. Also assume that the 4 professional terms are themselves colocated in their own semantic neighborhood. Assume that the controlled vocabulary resource contains 1 of the consumer terms and 1 of the professional terms. Also assume that the controlled vocabulary resource, according to its internally specified rules and structure, locates the contained consumer term and the contained professional term in the same semantic neighborhood. Finally, assume that the relationship between semantic neighborhoods is transitive, that is, if the consumer neighborhood may be linked to the controlled neighborhood, and the controlled neighborhood may be linked to the professional neighborhood, then the consumer neighborhood may be linked to the professional neighborhood. In that case the controlled vocabulary resource may be used to indirectly relate the remaining consumer and professional terms, thus providing a consumer entry vocabulary to the natural language professional vocabulary. The character of that relation will vary with the properties that define the respective semantic neighborhoods, as well as with the degree of control that characterizes the vocabulary resource.

In order to link natural language consumer terms to natural language professional terms we must, according to our model, group the consumer terms and professional terms into semantic neighborhoods ( **Methods, Step 3** ). In addition, the links between consumer terms and professional terms provided by a vocabulary resource are appropriate only if those terms should be linked. Thus, in order for us to evaluate whether a given vocabulary resource may be useful in linking natural language consumer terms to natural language professional terms, we need to independently determine whether the consumer terms should be colocated with the professional terms in the same semantic neighborhood ( **Methods, Step 3** ). Finally, while the conceptual appropriateness of the links provided by a vocabulary resource is important, we are most interested in the practical value of the resulting consumer entry vocabulary with respect to the previously mentioned goals of Healthy People 2010 and NCI. Thus, we need to evaluate the consumer entry vocabulary with respect to some context of information use or health care communication that can provide support for an individual's decision making about his or her personal health care. In this pilot study, we evaluated the consumer entry vocabulary for its support for Web retrieval of health care information ( **Methods, Step 8** ). Certainly, Web retrieval should be evaluated not only for the *amount* of information retrieved but also its *quality*. For this pilot study, we examined Web retrieval, using the entry vocabulary, for sites maintained by nonprofit health care professional organizations, academic organizations, or governmental organizations. We recognize that maintenance of a site by a professional, academic, or governmental organization does not guarantee that the site is of high quality, but we do think it does increase the likelihood that the site is of high quality.

To keep this initial exploratory study to a manageable size, we have restricted ourselves to consumer vocabulary related to diabetes, and have attempted to relate this consumer vocabulary to diabetes vocabulary used by US family physicians. As such, this study complements a previous study concerning consumer and professional vocabulary for diabetes that focused on the Read Thesaurus [35].

### Research Questions

We addressed 2 research questions:

1. To what extent can the Metathesaurus [39], the Eurodicautom of the European Commission's Translation Service [40], and the European Commission Glossary of popular and technical medical terms (or European Glossary for short) [41], as extended by the Dictionary of American Regional English (DARE) project [42-45], be used to derive pairs of consumer and physician terms concerning diabetes that are in the same semantic neighborhood?
2. Can retrieval of diabetes-related Web information sites maintained by nonprofit health care professional organizations, academic organizations, or governmental organizations be improved by substituting a physician term for its paired consumer term in a Web query?

### Vocabulary Resources

The Metathesaurus is a large database developed by the National Library of Medicine that links together terms from over 50 health care vocabularies. The Metathesaurus links terms together when they express the same, or nearly the same, concepts. When terms express the same concept, they are assigned, in the language of the Metathesaurus, to the same *metaconcept*. For example, the terms *Unspecified diabetes mellitus* and *Diabetes mellitus* are assigned to the same metaconcept.

The Eurodicautom is the multilingual terminological database of the European Commission's Translation Service. The Eurodicautom contains 124,551 entries for medicine, with many of these in English. Altogether, the Eurodicautom contains 990,672 English term entries. The Eurodicautom is organized by terminology collections maintained by different terminology offices, and, like the Metathesaurus, links different terms together when they express the same, or nearly the same, concepts. For example, the Eurodicautom entry for *hypertension,* in the terminology collection EUT97, includes *hyper blood pressure* as a synonym for *hypertension*.

The European Glossary was developed by the European Commission and is separate from the Eurodicautom. The European Glossary contains pairs of synonymous or nearly-synonymous consumer terms and professional terms. For example, the European Glossary pairs the consumer term holding your breath with the professional term hypoventilation.

DARE is intended to "document the varieties of English that are not found everywhere in the United States--those words, pronunciations, and phrases that vary from one region to another, that we learn at home rather than at school, or that are part of our oral rather than our written culture" [45]. DARE is based on fieldwork involving interviews carried out in 1,002 US communities in all 50 US states between 1965 and 1970. DARE fieldworkers collected a variety of responses to this interview question concerning diabetes, for example: "When a person has too much sugar in his blood and may have to take insulin for it, you'd say he has__________________?"

The responses included *diabetes*, *high blood*, *sugar blood*, *sugar diabetes*, and *the sugar*. Additional terms included in DARE are provided by a comprehensive collection of written materials (diaries, letters, novels, histories, biographies, newspapers, and government documents) that cover US history from the colonial period to the present [45].

## Methods

**Step 1.** We selected consumer terms related to diabetes from a list of terms previously extracted from 2 consumer sources. The first source of terms was a corpus of 1,500 e-mail questions submitted by consumers to University of Missouri Healthcare's On Call Online Web health care advice site during the period 1997-1999 (University of Missouri-Columbia Human Subjects Research Institutional Review Board [IRB] exemption approval #6843.) The second source of terms was a log of 348,000 queries submitted to the consumer portal of a major for-profit Web health care information site (by agreement with the site owner, we will not mention the name of the site.) The diabetes-related terms were selected by searching a list of previously-extracted terms for those containing the substrings *diab*, *dib*, *gluco*, or *insul*. We corrected the spelling of the consumer terms and eliminated duplicates. (We recognize at the outset a limitation of this study-that while these terms are ostensibly *consumer* terms, their pedigree as *layperson* terms must remain somewhat in doubt, since there is no clear evidence, particularly for terms extracted from the query log, to settle the question whether a health professional or a layperson entered the query.)

**Step 2.** We next extracted physician terms related to diabetes from terms previously extracted from a corpus of 25,000 family-medicine progress notes authored by University of Missouri Healthcare family physicians during the period 1999-2000 (University of Missouri-Columbia IRB exemption approval #7054.) As with the consumer terms, physician terms related to diabetes were selected by searching a list of previously extracted terms for terms containing the substrings *diab*, *dib*, *gluco*, or *insul*. We corrected the spelling of the physician terms and eliminated duplicates.

**Step 3.** Two co-authors (H.K.M. and T.B.P.) used the following informal procedure to produce pairs of consumer and physician terms that were judged to be in the same semantic neighborhood: (1) each consumer term was matched to one or more synonymous, nearly-synonymous, or closely-related physician terms; (2) the consumer terms and physician terms were separately grouped into sets of synonymous, nearly-synonymous, or closely-related terms; (3) each group of consumer terms was matched to one or more groups of physician terms; and (4) the consumer-term-physician-term matches were adjusted based on (3). H.K.M. did the initial matching and groupings of terms and then T.B.P. reviewed the matches and groupings. Disagreements were discussed and reviewed using medical dictionaries until consensus was achieved. This informal procedure was not intended to produce pairs of strictly-synonymous consumer terms and physician terms, but rather to produce pairs of terms we judged to be synonymous, nearly synonymous, or closely related. The approach was more informal and subjective than we would have liked. We would have preferred a method that would have allowed us to generate a logically-rigorous definition of the properties defining the semantic neighborhoods. However, objective measures of semantic locality , that would allow us to more-rigorously specify those properties, were not clearly applicable in this case. For example, we concluded that text corpus analysis such as word or term co-occurrence analysis [38,46] would not be applicable since we had no larger textual context for the set of terms extracted from the query log. The use of other empirical methods to establish semantic locality, such as focus groups, interviews, or surveys with laypersons and family physicians, was beyond the exploratory scope of this pilot study.

**Step 4.** We created extensions for the Metathesaurus, Eurodicautom, and the European Glossary with terms taken from DARE. We linked the DARE terms as a group to each vocabulary resource as near-synonyms of the general term *diabetes*. Even though there appear to be cases of folk-belief systems and associated vocabulary that are extremely precise in medical meaning [6], we adopted this conservative approach in order to avoid assigning inappropriately-specific meaning to the folk terms. However, this conservative approach may be inconsistent with the finding of [26] that in some cases persons who say they have *sugar* have very different health beliefs regarding their disease than do persons who say they have *sugar diabetes* or *diabetes*.

**Step 5.** Since we maintained the Metathesaurus and the European Glossary in local relational databases, we were able to easily automate searching of those databases. We used normalized string matches to search for the consumer terms and the physician terms in the DARE extensions of the Metathesaurus and the European Glossary. The normalized string associated with a term is produced by a rule-based transformation of the string of characters that carries the term, for example, the string of characters *eye doctors* carries the term *eye doctors*. According to the set of rules

1. Make plural singular
2. Put words in alphabetic order;

it may be normalized to the string *doctor eye*. Normalized string matches allow for more liberal term-to-term matches by preventing matches from being blocked by minor lexical variation. We created normalized-string indexes for the DARE extensions of the Metathesaurus and the European Glossary using the UMLS NORM program [39]. We then normalized the consumer and physician terms and matched them to strings in normalized-string indexes.

**Step 6.** The Eurodicautom is only available through a Web interface so we were not able to perform normalized string matches against it (according to an e-mail from J Vega J, official contact for questions and feedback about the Eurodicautom, 2001 Apr). Instead, we used the Eurodicautom interface to perform all-words-plus-context and truncate-only-if-no-match English-language searches against the Eurodicautom. In these searches, a term would match if all of its component words were contained in the Eurodicautom term. The use of truncation provides matches in cases of minor lexical variation. We created a table of DARE terms that we had linked to Eurodicautom terms, and a normalized string index. We searched the DARE extension of the Eurodicautom for the consumer and physician terms.

**Step 7.** We next collected pairs of consumer and physician terms that were contained either directly or indirectly in the DARE extensions of the 3 vocabulary resources. Some consumer and physician terms matched directly, via a string match, to a vocabulary resource, while some matched only indirectly by virtue of their semantic neighborhood. For example, the consumer term *diabetes diet* matched directly, while the consumer term *food for diabetics* only matched indirectly; *food for diabetics* matched indirectly because it was synonymous, nearly synonymous, or closely related to *diabetes diet*. We said that a consumer-term-physician-term pair was *directly covered* by a vocabulary resource when both of the following occurred:

- Both the consumer term and the physician term matched directly, via string matches, to the vocabulary resource
- The vocabulary resource, according to its internally-specified rules and structure, located the contained consumer term and the contained professional term in the same semantic neighborhood.

We considered the second condition satisfied for the Metathesaurus when either of the terms were associated with the same metaconcept, or were associated with distinct metaconcepts that are related according to the Metathesaurus related metaconcepts (MRREL) table. We considered the second condition satisfied for the Eurodicautom when the terms were matched to the same Eurodicautom entry - and similarly for the European Glossary.

A pair was *indirectly covered* by a vocabulary resource when both terms matched to terms in another pair that was directly covered, but at least one match was indirect. A vocabulary resource provided *partial indirect coverage* for a pair when only one member of the pair matched directly, and it provided *full indirect coverage* of the pair when neither member of the pair matched directly.

In order to provide preliminary results for our first research question, we compared the number of consumer and physician terms that directly and indirectly matched to the DARE extensions of the 3 resources, as well as the consumer-term-physician-term pairs that were directly and indirectly covered.

**Step 8.** In order to provide preliminary results for our second research question, coauthor T.B.P. selected 5 pairs that were provided partial-indirect coverage by the Metathesaurus, the Eurodicautom, or the European Glossary. For each of these pairs, the consumer term matched indirectly and the physician term matched directly. T.B.P. performed exact-phrase searches on www.altavista.com using the consumer term and physician term of each pair. Exact-phrase searches were used in order to better discriminate between the retrieval effects of the consumer term and the physician term. The results for the consumer term were compared to the results for the physician term. The results were compared for their respective numbers of relevant sites maintained by nonprofit health care professional organizations, academic organizations, or governmental organizations. Since anecdotal evidence suggests that consumers are likely to not look past the first few pages of results returned by a Web search engine, for any given search we examined only the first 20 results listed. We did not count a site if it was unavailable and we counted 2 or more pages from the same Web site as representing a single site.

## Results

### Results for research question 1

Our raw data consisted of 909 consumer terms (eg, *borderline diabetes*, *sugar dibetes*, *suger diabetes*) and 938 physician terms (eg, *insulin insensitivity* and *diabetic flow sheet*). We corrected spelling and merged duplicate terms. We then selected 86 consumer terms and matched them to 125 physician terms for a total of 225 pairs of terms. Table 1 shows example pairs of terms.

**Table 1:** Example Pairs of Consumer and Physician Terms

Consumer Term	Physician Term
diabetic food	healthy diabetic diet
diabetic food	diabetic diet plan
diabetes recipes	healthy diabetic diet
diabetes recipes	diabetic diet plan

We directly matched 27 consumer terms against the DARE extension of the Metathesaurus, 27 consumer terms against the DARE extension of the Eurodicautom, and 5 consumer terms against the DARE extension of the European Glossary. We indirectly matched 13 consumer terms against the DARE extension of the Metathesaurus, 11 consumer terms against the DARE extension of the Eurodicautom, and 1 consumer term against the DARE extension of the European Glossary.

We directly matched 39 physician terms against the DARE extension of the Metathesaurus, 23 physician terms against the DARE extension of the Eurodicautom, and 2 physician terms against the DARE extension of the European Glossary. We indirectly matched 29 physician terms against the DARE extension of the Metathesaurus, 7 physician terms against the DARE extension of the Eurodicautom, and no physician terms against the DARE extension of the European Glossary.

The DARE extension of the Metathesaurus directly covered 17 consumer-term-physician-term pairs, provided partial indirect coverage for 22 pairs, and full indirect coverage for 12 pairs. The DARE extension of the Eurodicautom directly covered 8 pairs, provided partial indirect coverage for 19 pairs, and full indirect coverage for 9 pairs. The DARE extension of the European Glossary directly covered 2 pairs, provided partial indirect coverage for 1 pair, and full indirect coverage for no pairs. Table 2 shows examples of directly and indirectly covered pairs of terms.

**Table 2:** Examples of Directly and Indirectly Covered Pairs of Terms

Consumer Term	Physician Term	Extended Metathesaurus	Extended Eurodicautom	Extended European Glossary
sugar diabetics	diabetes	partial indirect	partial indirect	partial indirect
sugar diabetes	diabetes	direct	direct	direct
diabetes	diabetes	direct	direct	direct
diabetes recipes	diabetic diet	partial indirect	partial indirect	not covered
diabetic food	healthy diabetic diet	full indirect	partial indirect	not covered
food to prepare for diabetic	healthy diabetic diet	full indirect	full indirect	not covered
diabetic diet	diabetic diet	direct	direct	not covered

### Results for Research Question 2

Table 3 compares the results of Web searches on www.altavista.com for the consumer terms and physician terms. The *Quality (out of first 20 results)* column under Results contains the number of sites out of the first 20 search results that were maintained by nonprofit health care professional organizations, academic organizations, or governmental organizations. In every case, the physician term produced results that contained more such sites than the consumer term.

**Table 3:** Comparison of Results of Consumer Term Searches and Physician Term Searches for Pairs with Partial Indirect Coverage

Consumer Term			Physician Term			Vocabulary Resource Providing Partial Indirect Coverage
Term	Results		Term	Results		
	Total	Quality* (out of first 20 results)		Total	Quality* (out of first 20 results)	
diabetes recipes	457	1	diabetic diet	83,778	2	Metathesaurus; Eurodicautom
foods for diabetics	100	0	diabetic diet	83,778	2	Metathesaurus; Eurodicautom
diabetes in children	2,016	1	juvenile diabetes	18,482	6	Eurodicautom
diabetic pregnancy	562	0	gestational diabetes	13,498	2	Metathesaurus
sugar diabetics	92	0	diabetes	1,038,268	6	Metathesaurus; Eurodicautom; European Glossary
organizations						

## Discussion

The results for our first research question for the DARE extension of the Metathesaurus appear somewhat promising. The DARE extension of the Metathesaurus provided coverage, either direct or indirect, for approximately 23% of the natural language consumer-term-physician-term

pairs. The results provided by the Eurodicautom extension are less promising, since it provided coverage for only 16% of the term pairs. (The results for the European Glossary were negligible.) However, what we find somewhat promising overall is that both the Metathesaurus and the Eurodicautom extensions indirectly covered more pairs than they directly covered. A high percentage of covered term pairs, with more indirectly-covered pairs than directly-covered pairs, might constitute an efficient use of expensive controlled vocabulary resources for health care communications.

The results for the second research question are also somewhat promising. Notwithstanding the small sample size, in every case the natural language physician term produced better results than the consumer term for sites maintained by nonprofit health care professional organizations, academic organizations, or governmental organizations. This suggests that the DARE extensions of the Metathesaurus and Eurodicautom may be used to provide useful links from natural language consumer terms to natural language physician terms. It might be argued that these results are not particularly meaningful since

We performed our study using terms rather than actual consumer queries

Maintenance of a site by a nonprofit health care professional, academic, or governmental organization is not a certain indicator of the quality of the site.

In response to the first point, we point out that some of the terms we used in our www.altavista.com queries were extracted from longer consumer e-mail messages, and some were used as actual queries to a Web information site. These terms reflect the actual terms used by consumers, although admittedly only the latter can be said to represent actual consumer Web queries (subject to the limitation noted earlier in **Methods, Step 1** , concerning their pedigree as *layperson* terms). It is also true that we corrected the spelling of some consumer terms. However, the effect of misspellings on retrieval is not clear and is a subject of our current investigations. Finally, many Web search programs that accept natural language input extract terms from it and use them for retrieval as we did in our study.

In response to the second point, we agree that maintenance of a site by a professional, academic, or governmental organization does not guarantee that the site is of high quality, but, as stated in **Overview of the study** , we do think such maintenance increases the likelihood of high quality.

### Limitations of the study

Although our results are somewhat promising, they are limited in several ways. One limitation of our study is that www.altavista.com, like most search engines, does not limit its exact-phrase results to results for true exact phrases, but will also report results where the words in the phrase are included in the document, but are not immediately next to each other. Thus, our results do not strictly discriminate between the retrieval effectiveness of the consumer terms and the physician terms. Our results do, however, reflect the situation actually faced by consumers, and to that extent are indicative of the relative effectiveness of the consumer and physician terms.

Another limitation of the study is that one of our sources of consumer terms, the Web query log, did not lend itself to term co-occurrence as a quantifiable measure of semantic locality, nor to other data that might be used to cluster terms into semantic neighborhoods. But, perhaps more importantly, we did not evaluate the final term pairs with respect to other quantitative evidence derived from consumer and physician surveys, nor did we evaluate them with respect to qualitative evidence derived from focus groups and interviews with consumers and physicians. Thus, even if the term pairs appear useful according to the standard of quality that we used in this project, they might not be more generally useful, because they might not constitute appropriate meaning-preserving (or meaning-warping) steps from the language of consumers to the language of family physicians.

Finally, although this study may serve in part as a pilot for larger studies of access to consumer health care information, a general limitation of the study is that we focused on the Web, and did not address the use of other venues of medical information, such as newspapers, magazines, and television. We recognize that these other sources and formats of health information require similar investigation if consumers are to get the information they need to support their decisions about their personal health care. Indeed, as we said at the outset, the bidirectional communication problems we have been considering are more fundamental than the Web and the digital divide. More work needs to be done with physicians, patient-education professionals, and consumers to further articulate the extent of these problems and to develop methods for their resolution.

## Abbreviations

AP: Associated Press
CDC: Center for Disease Control
DARE: Dictionary of American Regional English
IRB: Institutional Review Board
NCI: National Cancer Institute
UMLS: Unified Medical Language System
US: United States

## References

[1] United States Dept. of Health and Human Services (2000). Healthy People 2010: Understanding and Improving Health.

[2] National Cancer Institute Centers of excellence in cancer communications research. Publication RFA-CA-01-019.

[3] National Cancer Institute Scientific priorities for cancer research: extraordinary opportunities.

[4] Taylor H (2000). The Harris Poll #44: Explosive growth of "cyberchondriacs" continues.

[5] Brodie M, Flournoy R E, Altman D E, Blendon R J, Benson J M, Rosenbaum M D (2000). Health information, the Internet, and the digital divide. Health Aff (Millwood).

[6] Burnum J F (1984). Dialect is diagnostic. Ann Intern Med.

[7] Snow L F (1974). Folk medical beliefs and their implications for care of patients. A review bases on studies among black Americans. Ann Intern Med.

[8] Sugarman J, Butters R R (1985). Understanding the patient: medical words the doctor may not know. N C Med J.

[9] Duffy F D (1998). Dialogue: the core clinical skill. Ann Intern Med.

[10] Salmon P, Peters S, Stanley I (1999). Patients' perceptions of medical explanations for somatisation disorders: qualitative analysis. BMJ.

[11] Skelton J R, Hobbs F D (1999). Descriptive study of cooperative language in primary care consultations by male and female doctors. BMJ.

[12] Steel N (1999). Communicating with patients. Specialist training should include communication skills. BMJ.

[13] Freeman J, Loewe R (2000). Barriers to communication about diabetes mellitus. Patients' and physicians' different view of the disease. J Fam Pract.

[14] Kefalides P T (1999). Illiteracy: the silent barrier to health care. Ann Intern Med.

[15] Falvo Donna R (1994). Effective Patient Education: a Guide to Increased Compliance.

[16] Leichter S B, Nieman J A, Moore R W, Collins P, Rhodes A (1981). Readability of self-care instructional pamphlets for diabetic patients. Diabetes Care.

[17] Reid J C, Klachko D M, Kardash C A, Robinson R D, Scholes R, Howard D (1995). Why people don't learn from diabetes literature: influence of text and reader characteristics. Patient Educ Couns.

[18] Hardie G E, Janson S, Gold W M, Carrieri-kohlman V, Boushey H A (2000). Ethnic differences: word descriptors used by African-American and white asthma patients during induced bronchoconstriction. Chest.

[19] Weiland S K, Kugler J, Von Mutius E, Schmitz N, Fritzsch C, Wahn U, Keil U (1993). [The language of pediatric asthma patients. A study of symptom description]. Monatsschr Kinderheilkd.

[20] Mahler D A, Harver A (2000). Do you speak the language of dyspnea?. Chest.

[21] Moy M L, Lantin M L, Harver A, Schwartzstein R M (1998). Language of dyspnea in assessment of patients with acute asthma treated with nebulized albuterol. Am J Respir Crit Care Med.

[22] Flynn C A, Barash A (2000). Do African American asthmatics perceive and describe their asthma symptoms differently than white asthmatics?. J Fam Pract.

[23] Hornberger J, Itakura H, Wilson S R (1997). Bridging language and cultural barriers between physicians and patients. Public Health Rep.

[24] Woloshin S, Bickell N A, Schwartz L M, Gany F, Welch H G (1995). Language barriers in medicine in the United States. JAMA.

[25] Texas Cancer Council Practical guidelines for the development of audiovisual cancer education materials for African Americans.

[26] Schorling J B, Saunders J T (2000). Is "sugar" the same as diabetes? A community-based study among rural African-Americans. Diabetes Care.

[27] Sievert M E, Patrick T B, Reid J C (2001). Need a bloody nose be a nosebleed? or, lexical variants cause surprising results. Bull Med Libr Assoc.

[28] Associated Press (2001). Ferraro battling blood cancer. Columbia Daily Tribune 2001.

[29] Health Summit Working Group Criteria for assessing the quality of health information on the Internet - policy paper.

[30] Wootton J C (1997). The quality of information on women's health on the Internet. J Womens Health.

[31] Oermann M H, Wilson F L (2000). Quality of care information for consumers on the Internet. J Nurs Care Qual.

[32] Adelhard K, Obst O (1999). Evaluation of medical internet sites. Methods Inf Med.

[33] Lancaster F Wilfrid (1986). Vocabulary Control for Information Retrieval.

[34] Brown P J, Price C, Sonksen P H (1997). Evaluating the terminology requirements to support multi-disciplinary diabetes care. Proc AMIA Annu Fall Symp.

[35] Brown PJB, Price C, Cox YM (1997). Patient language - evaluating its relationship to a clinical thesaurus. Proc AMIA Annu Fall Symp.

[36] Buckland M, Chen A, Kim Y, Lam B, Larson R (1999). Mapping Entry Vocabulary to Unfamiliar Metadata Vocabularies. D-Lib Magazine.

[37] Jones James H (1993). Bad BLood: The Tuskegee Syphilis Experiment, Revised Edition.

[38] Bodenreider O, Nelson S J, Hole W T, Chang H F (1998). Beyond synonymy: exploiting the UMLS semantics in mapping vocabularies. Proc AMIA Symp.

[39] National Library of Medicine (2000). UMLS Knowledge Sources.

[40] European Commission's Translation Service Eurodicautom.

[41] European Commission Multilingual glossary of technical and popular medical terms in nine European languages.

[42] Cassidy FG (1985). Dictionary of American Regional English.

[43] Cassidy FG (1991). Dictionary of American Regional English.

[44] Cassidy FG (1996). Dictionary of American Regional English.

[45] DARE Dictionary of American Regional English.

[46] Biber Douglas, Conrad Susan, Reppen Randi (1998). Corpus Linguistics : Investigating Language Structure and Use (Cambridge Approaches to Linguistics).

